# *N*-acetylglucosaminyltransferases and nucleotide sugar transporters form multi-enzyme–multi-transporter assemblies in golgi membranes in vivo

**DOI:** 10.1007/s00018-019-03032-5

**Published:** 2019-02-08

**Authors:** Fawzi Khoder-Agha, Paulina Sosicka, Maria Escriva Conde, Antti Hassinen, Tuomo Glumoff, Mariusz Olczak, Sakari Kellokumpu

**Affiliations:** 1 Faculty of Biochemistry and Molecular Medicine, University of Oulu, Aapistie 7A, 90220 Oulu, Finland; 2 Laboratory of Biochemistry, Faculty of Biotechnology, University of Wroclaw, Wrocław, Poland; 3 Present Address: Human Genetics Program, Sanford Burnham Prebys Medical Discovery Institute, La Jolla, CA USA; 4 Present Address: Faculty of Biology, University of Barcelona, Barcelona, Spain; 5 Present Address: Institute of Molecular Medicine, Helsinki, Finland

**Keywords:** Golgi apparatus, Enzyme complexes, Membrane organization, Glycosylation

## Abstract

Branching and processing of *N*-glycans in the medial-Golgi rely both on the transport of the donor UDP-*N*-acetylglucosamine (UDP-GlcNAc) to the Golgi lumen by the SLC35A3 nucleotide sugar transporter (NST) as well as on the addition of the GlcNAc residue to terminal mannoses in nascent *N*-glycans by several linkage-specific *N*-acetyl-glucosaminyltransferases (MGAT1-MGAT5). Previous data indicate that the MGATs and NSTs both form higher order assemblies in the Golgi membranes. Here, we investigate their specific and mutual interactions using high-throughput FRET- and BiFC-based interaction screens. We show that MGAT1, MGAT2, MGAT3, MGAT4B (but not MGAT5) and Golgi alpha-mannosidase IIX (MAN2A2) form several distinct molecular assemblies with each other and that the MAN2A2 acts as a central hub for the interactions. Similar assemblies were also detected between the NSTs SLC35A2, SLC35A3, and SLC35A4. Using in vivo BiFC-based FRET interaction screens, we also identified novel ternary complexes between the MGATs themselves or between the MGATs and the NSTs. These findings suggest that the MGATs and the NSTs self-assemble into multi-enzyme/multi-transporter complexes in the Golgi membranes in vivo to facilitate efficient synthesis of complex *N*-glycans.

**Electronic supplementary material:**

The online version of this article (10.1007/s00018-019-03032-5) contains supplementary material, which is available to authorized users.

## Introduction

Glycosylation is the most common modification of proteins and lipids, and crucial for multicellular life given its roles in a plethora of basic cellular functions. For example, glycosylation helps proteins to fold correctly, ensures their optimal transport to the cell surface, and mediates a variety of interactions between cells, cells and the extracellular matrix and certain pathogens that use glycans as specific recognition and attachment sites. Therefore, glycosylation is under strict control to allow faithful synthesis of thousands of structurally unique and functionally important glycans on glycoproteins and glycolipids.

Processing of high mannose *N*-glycans to complex *N*-glycans commences in the cis/medial-Golgi. It relies on the activity of several distinct *N*-acetylglucosaminyltransferases termed MGAT1, MGAT2, MGAT3, MGAT4A,4B, MGAT5A,5B and possibly MGAT6 [37] that each add *N*-acetylglucosamine (GlcNAc) from UDP-GlcNAc to a specific mannose in the core of a *N*-glycan. In brief, MGAT1 adds the first GlcNAc residue to the C-2 of the α1-3Man in the core of Man_5_GlcNAc_2_. Once this step takes place, α-mannosidase II (MAN2A1 or MAN2A2) removes the terminal α1-3Man and α1-6Man residues to form a GlcNAcMan_3_GlcNAc_2_*N*-glycan. Consequently, MGAT2 adds a GlcNAc residue to the C-2 of the α1-6Man, paving the way for additional branching to generate tri- and tetra-antennary *N*-glycans at C-4 of the core α1-3Man and at C-6 of the core α1-6Man by MGAT4A (or MGAT4B) and MGAT5A (or MGAT5B), respectively.

The substrate required in these processes is UDP-*N*-acetylglucosamine (UDP-GlcNAc). Given the synthesis of UDP-GlcNAc and other nucleotide sugars in the cytosol [4] (or in the nucleus in the case of the CMP-sialic acid [22]), they need to be delivered across the Golgi/ER membrane by relevant nucleotide sugar transporters (NSTs) [2]. These transporters are multi-spanning transmembrane proteins that act as antiporters by exchanging the nucleotide sugar with the corresponding nucleotide monophosphate. The latter is produced following the transfer of a monosaccharide by the relevant glycosyltransferase (GTase) [3] and the decomposition of the relevant NDP to NMP and inorganic phosphate by nucleotide diphosphatases (NDPases) [6].

Although GTases and NSTs are often presented as independent players in glycosylation, recent evidence indicates that the enzymes and transporters are functionally organized into highly specific and dynamic complexes both in yeast, plants and mammalian cells [5, 7, 8, 15, 26, 30, 34, 37, 38]. Complex formation for the GTases involves both homomeric and heteromeric interactions, the latter occurring selectively between sequentially acting enzymes within each glycosylation pathway. The formation of enzyme heteromers appears to be strictly dependent on, and regulated by, Golgi micro-environmental factors such as pH (ion) homeostasis [9]. Enzyme heteromers appear also to be functionally more important than the homomers, as their formation increases their enzymatic activity [9–11, 15]. Enzyme interactions are also mediated mainly by direct contacts between their catalytic domains [11], a phenomenon that is anticipated to increase both the efficiency and accuracy of glycan biosynthesis, e.g. via prevention of intervention by competing enzymes [5]. Such GTase heteromers have been detected in all major glycosylation pathways and in the different Golgi sub-compartments [15]. Of these, the medial Golgi GTases have been shown to form mega-dalton size assemblies [28], yet specific interactions have been demonstrated separately only between MGAT1, MGAT2 and an alpha-mannosidase II [24, 25].

NSTs similarly form homomeric complexes (either dimers or higher-order homo-oligomers) in the Golgi compartment. For example, homomeric transporter assemblies have been detected in the case of a UDP-*N*-acetylgalactosamine transporter [32], a GDP-fucose transporter [31], a GDP-mannose transporter [1, 14, 29], a UDP-galactose transporter (SLC35A2) [27] and UDP-*N*-acetylglucosamine transporter (SLC35A3) [19]. Structural data on a GDP-mannose transporter also support its tendency to homo-oligomerize [29]. NSTs have also been shown to form heteromeric complexes with each other. For example, the UDP-GlcNAc and UDP-Gal transporters were found to interact in vivo [19]. Very recently, it was shown that MGAT1, MGAT2, MGAT4B and MGAT5 can also separately interact with both a UDP-GlcNAc transporter and a UDP-Gal transporter [18, 20].

To understand better how the GTases and NSTs are functionally organized in the Golgi membranes, we addressed here in detail mutual interactions between the medial-Golgi MGATs, or the NSTs, or those between the MGATs and the NSTs. In the study, we utilized FRET, BiFC and BiFC-based FRET high-throughput interaction screens that allow parallel analyses of multiple protein–protein interactions including ternary ones in thousands of cells in a single experiment. Our data reveal several novel and specific MGAT or NST assemblies in the Golgi membranes of live cells. For the first time, we were also able to identify ternary complexes between the MGATs or between the MGATs and the NSTs. Our findings thus provide direct evidence for the existence of multi-enzyme/multi-transporter assemblies in the Golgi membranes that not only may suffice for Golgi retention, but also to facilitate efficient branching and processing of high-mannose *N*-glycans to complex *N*-glycans.

## Materials and methods

### Construction of expression plasmids

All the plasmids that were used in this study are schematically presented in supplementary Fig. 1 (Fig. S1). Detailed information regarding the cloning strategies, the primer sequences and sequences of the constructs is available upon request.

**Fig. 1:**
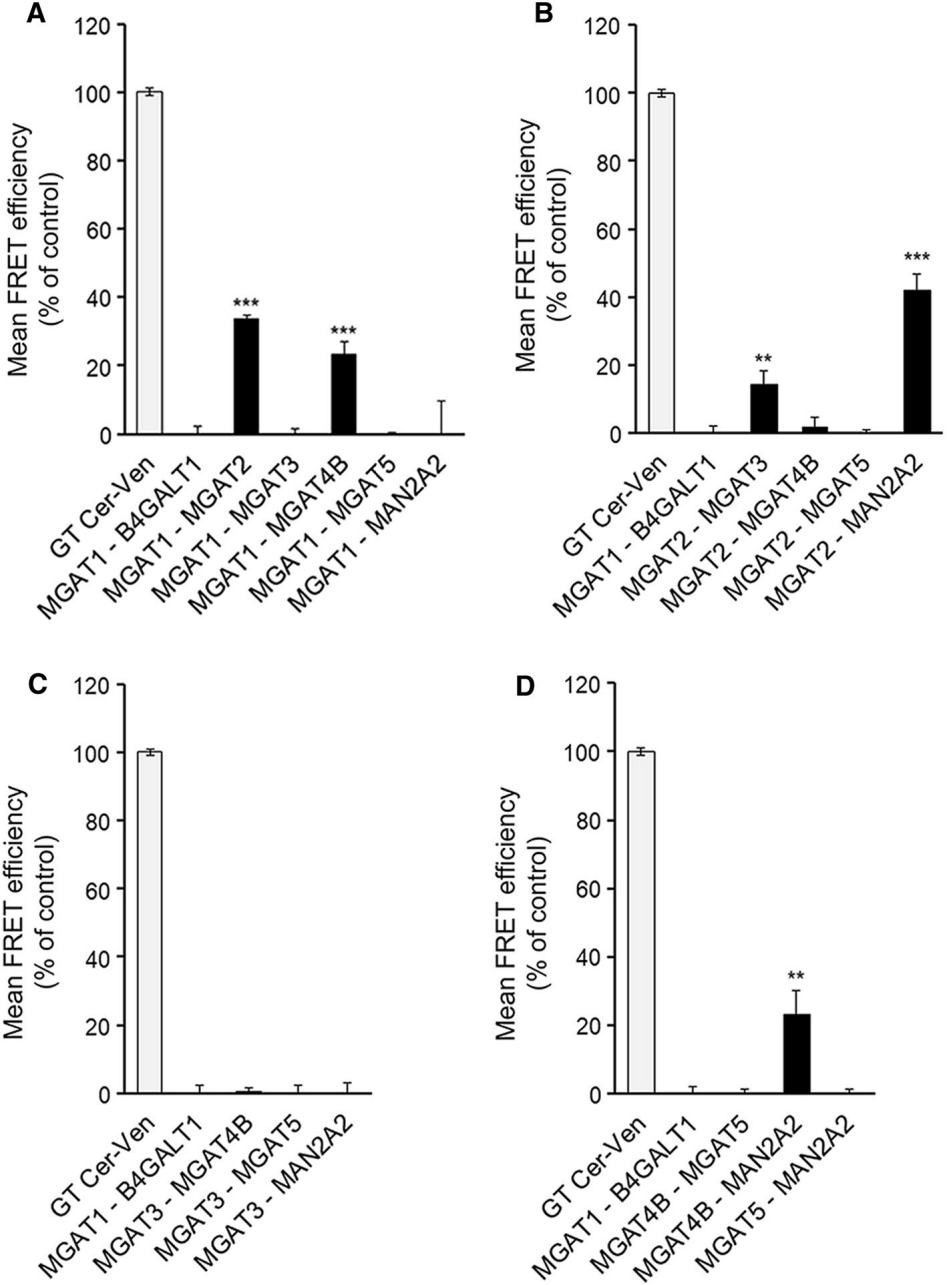
Mutual interactions between the medial-Golgi MGATs. A. MGAT1. B. MGAT2. C. MGAT3. D. MGAT4B (as well as MAN2A2 and MGAT5). COS7 cells were transfected with the depicted m-Cerulean- and mVenus-tagged FRET enzyme constructs, fixed 24 h later and quantified with the Operetta High Content Imaging system. FRET efficiencies were calculated and expressed as percentages of control values (mean ± SD, *n* = 3) after subtracting the FRET efficiencies of negative control (MGAT1-B4GALT1) values

### Cell maintenance and transfections

COS-7 cells were grown in Dulbecco’s modified Eagle’s medium (DMEM) supplemented with 10% fetal bovine serum, 4 mM l-glutamine, 100 U of penicillin/ml and 100 µg of streptomycin/ml. COS-7 cells were transiently transfected with expression plasmid(s) using Fugene6™ (Promega) according to the manufacturer’s instruction as described elsewhere [10, 11]. For BiFC analysis, cells were subjected to the immunofluorescence staining after 18 h of the transfections. For FRET and BiFC-based FRET analyses, cells were seeded onto 96-well plates (Ultra Perkin Elmer). 18 h post-transfection. After additional 5 h, cells were fixed for 15 min with 4% paraformaldehyde in PBS buffer, washed with PBS before the FRET measurements.

### FRET interaction assays

The FRET assay system is an energy transfer system dependent on the distance between one fluorescent donor and one fluorescent acceptor molecule. In each case, the two fluorophores are selected based on their emission and excitation spectra so that the donor’s emission spectrum overlaps with the acceptor excitation spectrum. When the donor molecule is excited, and if the donor (emission) is sufficiently close to the acceptor, an energy transfer will take place, resulting in generation of the emission signal from the acceptor, indicating a positive interaction. At the same time, the emission from the donor fluorophore decreases due to energy transfer to an acceptor fluorophore. If the donor is sufficiently far away from the acceptor (> 10 nm), then energy transfer cannot take place and no signal will be emitted, indicating a negative interaction.

For all FRET measurements, cells were transfected with Fugene6™ with the relevant FRET plasmids (see below), 18 h later seeded onto Cell Carrier Ultra™ 96-well plates, and allowed to attach for 6 h, before fixing with 4% paraformaldehyde (PFA) and washing with PBS (pH 7.4) [9]. In the present study, we used two fluorophore FRET pairs; mCerulean-mVenus and mVenus-mCherry. Using Operetta™ High-Through Put Imaging System (Perkin-Elmer Inc.), we measured first the total emission signal of each fluorophore when excited with their respective wavelengths (mCerulean exc 425–450, em 460–500; mVenus exc 460–490, em 500–550; mCherry exc 560–580, em 590–640). Thereafter, we measured the acceptor emission signal (FRET signal) when exciting only the donor (mCer-mVen pair: exc 425–450, em 500–550 or mVen-mChe pair: exc 460–490, em: 590–640). The data values obtained from these measurements were then used to determine the background fluorescence level of the FRET channel (less than 200 intensity units). We also determined total fluorescence signals for each channel to allow normalization and the calculation of their leakage rates (as %, also see the formula below) between different channels. The raw total data values (mean intensity 1000-2000 units) thus are only 5–10 times higher than the background autofluorescence signal. Due to the relatively small size of the Golgi and to evaluate the specificity of such interactions, we co-expressed Golgi localizing enzymes (such as MGAT1 and B4GalT1 or MGAT1 and ST6Gal1) that do not interact and used them as negative controls. In addition, to confirm that the fluorescence signals are measured only from the Golgi area, and not from the ER or other organelles, we opted the segmentation protocol built in “Harmony 4.1” software (Perkin-Elmer Inc.) that relied on using mVenus as a marker. Harmony also allows to select the exact Golgi area by making fine adjustments to the area size, the contrast of mVenus, common and individual threshold values as well as the split factor. An additional step of filtering was added as part of “select population” command to remove any areas that do not conform to set standard Golgi dimensions and intensity values below 400 (background). Furthermore, to remove cells with extremely high over-expression, we added an upper intensity limit for mVenus (< 8000 intensity unit) for the cell selection protocol. In addition, to exclude any signal or hue coming from the ER, we assigned borders around the Golgi area (10 pixels for the outer border and 5 pixels for the inner border) to subtract this signal from the Golgi-associated raw data values. This maneuver was used as a strict and stringent step to ensure the specificity of the numbers coming solely from the Golgi-localized enzyme constructs. Finally, to generate corrected FRET signals, we utilized the Youvan formula as follows:

$$\begin{aligned} & {\text{FRET}}\;{\text{signal }}\left( {{\text{mVenus}}\, - \,{\text{mCherry}}\,{\text{pair}}} \right) \\ & \quad = {\text{IF}}\left( {\text{FRET}} \right)\, - \,\left( {0. 10 2 6\, \times \,{\text{IF}}\left( {\text{mVenus}} \right)} \right) + \left( {0.0 60 6\, \times \,{\text{IF}}\left( {\text{mCherry}} \right)} \right), \\ \end{aligned}$$

or,

$$\begin{aligned} & {\text{FRET}}\;{\text{signal}}\,\left( {{\text{mCerulean}}\, - \,{\text{mVenus}}\,{\text{pair}}} \right) \\ & \quad = {\text{IF}}\left( {\text{FRET}} \right)\, - \,\left( {0. 8 8 8\; \times \;{\text{IF}}\left( {\text{mCerulean}} \right)} \right)\, + \,\left( {0.0 7 7 3\, \times \,{\text{IF}}\left( {\text{mVenus}} \right)} \right). \\ \end{aligned}$$

The IF in the formula denotes fluorescence intensity of the mVenus, mCherry and mCerulean channels, and the numbers correspond to the pre-determined leakage (as %) into the FRET channel from mVenus, mCherry and mCerulean channels, when excited using excitation wavelengths for mVenus or mCerulean. The corrected FRET data values were then converted to FRET efficiency percentages using the formula:

$$\frac{{{\text{corrected}}\;{\text{FRET}}\;{\text{acceptor}}\;{\text{intensity}}\, \times \, 100}}{{ \left( {{\text{corrected}}\;{\text{donor}}\;{\text{intensity}}\, + \,{\text{corrected}}\,{\text{FRET}}\,{\text{intensity}}} \right)}}.$$

### Bimolecular fluorescence complementation (BiFC) and indirect immunofluorescence

Cells were transfected with relevant BiFC enzyme constructs tagged N-terminally with non-fluorescent mVenus fragments (VN and VC), and C-terminally with the HA epitope tag or the FLAG tag and subjected to immunofluorescence staining 18 h post-transfection. To confirm the expression and Golgi localization of the different non-fluorescent VN and VC fusion constructs, we applied the previously described staining protocol [16, 33]. N-terminal VN fusion proteins were detected using the C-terminal HA-epitope tag antibody (monoclonal mouse anti-HA, 1:500 dilution, Sigma-Aldrich), whereas the VC fusion proteins were detected using the rabbit anti-FLAG antibodies (1:250 dilution, Sigma-Aldrich). As secondary antibodies, goat anti-mouse conjugated with Alexa Fluor 405 and goat anti-rabbit conjugated with Alexa Fluor 594 (1:500 dilution; Molecular Probes) were employed. After staining, cells were imaged using the Zeiss LSM700 confocal unit attached to Axio Observer.Z1 microscope, 63 × PlanApo oil immersion objective, and appropriate filter sets for Cerulean, YFP and Alexa Fluor 594 fluorophores. The BiFC signals were quantified from single and double transfected cells using the Operetta High-Content Imaging System. Only cells that expressed either the VN and VC (identified by anti-HA- and anti-FLAG antibodies, respectively) or both VN and VC fusion proteins were used for the quantification.

### BiFC-based FRET approach

To enable detection of three separate partners simultaneously, we utilized the BiFC-based FRET approach. In brief, cells were transfected with relevant VN, VC and mCherry- or mCerulean-enzyme encoding plasmid constructs, seeded onto the Cell Carrier Ultra™ 96-well plates 18 h post-transfection, and allowed to attach for 5 h before fixation. Data acquisition and quantification were performed with Operetta High-Content Imaging System similarly to that described above.

### Statistical analyses

Statistical significance of the interaction data sets (with at least three replicates and 10–20 × 10^3^ cells/experiment) were evaluated using either the one-way analysis of variance (ANOVA) (*p* ≥ 0.05 ns; *p < 0.05; **p < 0.01; ***p < 0.001) or the one tailed *t* test (*p* ≥ 0.05 ns; *p < 0.05; **p < 0.01; ***p < 0.001). All statistical analyses were performed using Microsoft Excel software.

## Results

### MGATs interact with each other in a highly specific manner

**Fig. 2:**
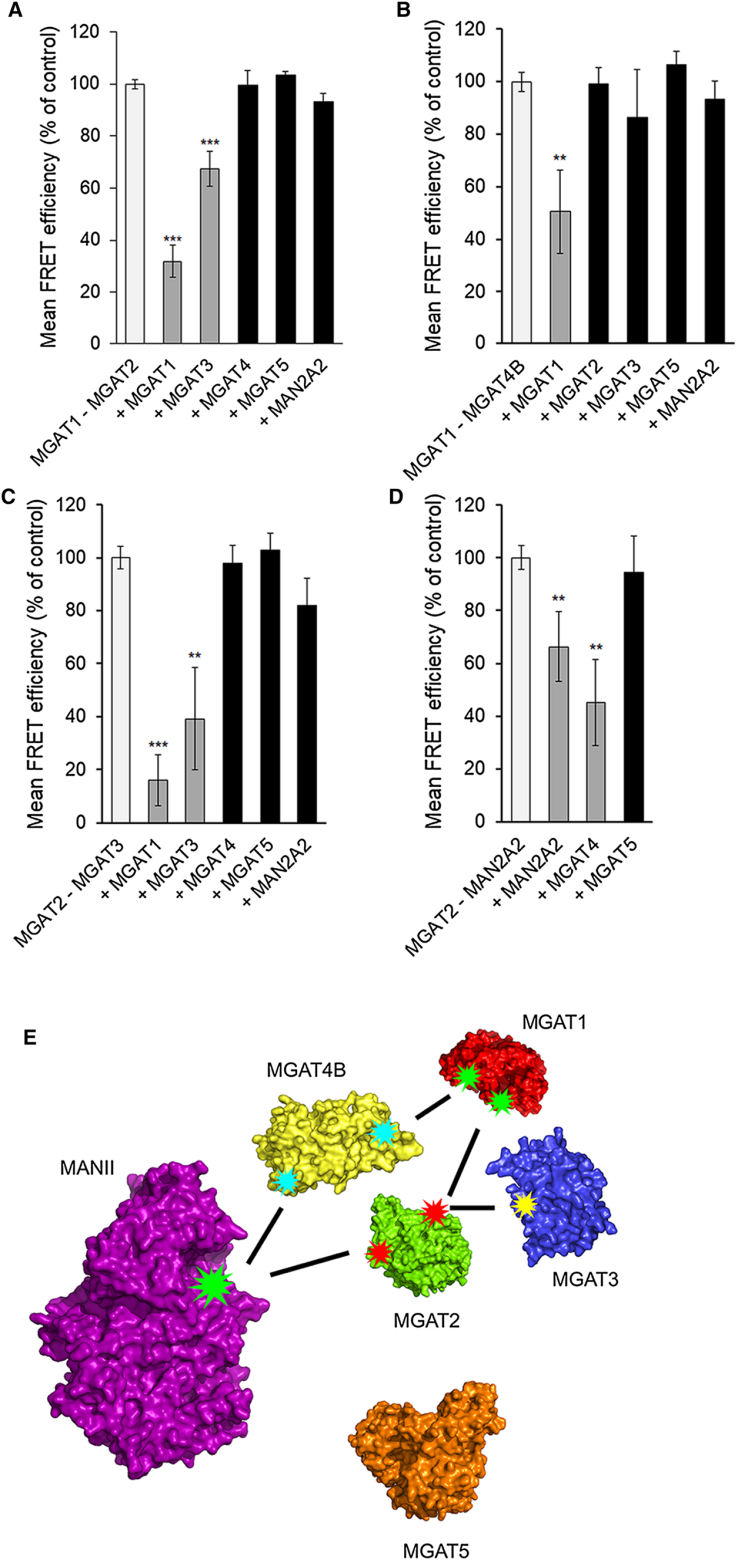
Inhibition of detected MGAT interactions with competing HA-tagged MGAT enzyme constructs. **a** MGAT1/MGAT2 interaction. **b** MGAT1/MGAT4B interaction. **C**. MGAT2/MGAT3 interaction. **d** MGAT2/MANII (MAN2A2) interaction. Cells were triple-transfected using HA-tagged competing constructs together with the depicted FRET pair (left column) in each graph. 24 h later, cells were fixed before quantification of the FRET signal with Operetta High Content Imaging System and calculation of the FRET efficiencies. The data are expressed as percentages (± SD, *n* = 3) of the control values (set to 100%; no competing construct present). (**e**) A curated interaction map based on the inhibition data above. Each star represents a single binding interface on each enzyme. Lines pointing to the same interaction surface denote competing interactions between the enzymes. The structures shown were obtained using existing atomic coordinates present in the PDB database. The following ID numbers were used: 2am3 (MGAT1), 5vcm (MGAT2), 5zic (MGAT5). MGAT3 and MGAT4B structures were modelled by ModBase

Interactions between the different MGATs were investigated using the FRET (Förster fluorescence resonance energy transfer) approach. The FRET approach relies on the detection of the FRET signal emitted by the acceptor fluorophore after excitation of the donor fluorophore only when the two fluorophores are less than 10 nm from each other. To accomplish this, we co-transfected cells with the plasmid encoding one MGAT-monomeric Cerulean (mCer, donor fluorophore) alone or together with a plasmid encoding each of the other MGATs or MAN2A2 tagged with the mVenus (mVen) acceptor fluorophore. As a positive control, we used a plasmid encoding mCer and mVen fluorophores fused together with a short linker region and including a Golgi targeting sequence derived from the N-terminus of β1,4-galactosyltransferase I (B4GALT1) [10]. This construct served as an internal control for FRET measurements and allowed normalization of the FRET signals acquired in different experiments. As a negative control, we co-expressed MGAT1-mCer with B4GalT1-mVen, a transferase that adds Gal to *N*-glycans and is topologically identical to MGATs. Its use enabled us to discriminate true FRET signal from the background before calculating FRET efficiencies (%), and to exclude non-specific FRET signal that might arise due to overexpression of the FRET constructs. We found that MGAT1 interacted with MGAT2 (as expected) and also with MGAT4B, but not with MGAT3, MGAT5 or MAN2A2 (Fig. 1a). MGAT2, besides interacting with MGAT1, also interacted with MGAT3 and MAN2A2 (Fig. 1b). However, it did not with either MGAT4B or MGAT5. Similarly, MGAT3 was found to interact only with MGAT2 (Fig. 1b, c). On the other hand, MGAT4B was found to interact with both MGAT1 (Fig. 1a) and MAN2A2 (Fig. 1d). Intriguingly, however, we did not detect any interactions between MGAT5 and the other MGATs (Fig. 1a–d) in the same assay conditions. Collectively, these data show that each MGAT (except MGAT5) has specific and defined MGAT partners in the medial-Golgi. These data are thus in contrast to what one can expect if the interactions are non-specific and driven by overexpression of the constructs. To assess in more detail the specificity and competitive nature of the interactions (i.e. whether enzymes can simultaneously interact with each other), we performed inhibition assays by transfecting cells with the selected FRET pair constructs together with an HA-tagged MGAT construct. We found that the MGAT1–MGAT2 interaction (Fig. 2a) was significantly inhibited by MGAT1 (as expected, given that it is part of the same complex), but also by MGAT3, while MGAT4B, MGAT5 or MAN2A2 had no effect. This is consistent with MGAT3 binding to MGAT2 (Fig. 1b) via the same interface than that used by MGAT1 (see also the interaction map in Fig. 2e). On the other hand, the MGAT1–MGAT4B interaction (Fig. 1a) was inhibited by MGAT1 (as expected), but not by MGAT2 (Fig. 2b), suggesting that MGAT1 binds to MGAT4B via an interface that is distinct from the one it uses for MGAT2 binding (see also Fig. 2e). Thus, MGAT1, MGAT2 and MGAT4B may form a trimeric complex together. The MGAT2–MGAT3 interaction (Fig. 1b) was in turn inhibited by both MGAT1 and MGAT3 (Fig. 2c), but not by MGAT4B, MGAT5 or MAN2A2. This suggests that MGAT1 and MGAT3 compete for binding to the same interface in MGAT2. Finally, the interaction between MGAT2 and MAN2A2 (Fig. 1b) was inhibited by MAN2A2 as expected, but also by MGAT4B (Fig. 2d). Thus, MGAT4B interacts with MAN2A2 via the same interface than that used by MGAT2 (Fig. 2e), indicating that the suggested ternary complex between MGAT1, MGAT2 and MGAT4B is not possible and cannot exist. In all tested cases, MGAT5 did not inhibit any of the detected interactions, consistent with its inability to interact with the other MGATs or with MAN2A2. These inhibition studies, besides conforming to the specificity of the detected interactions, show that MGATs in general form specific sub-complexes with each other and compete for binding with Man2A2. MGAT 5 appears to be an orphan MGAT and does not seem to interact with any other MGAT.

### Nucleotide-sugar transporter interactions

**Fig. 3:**
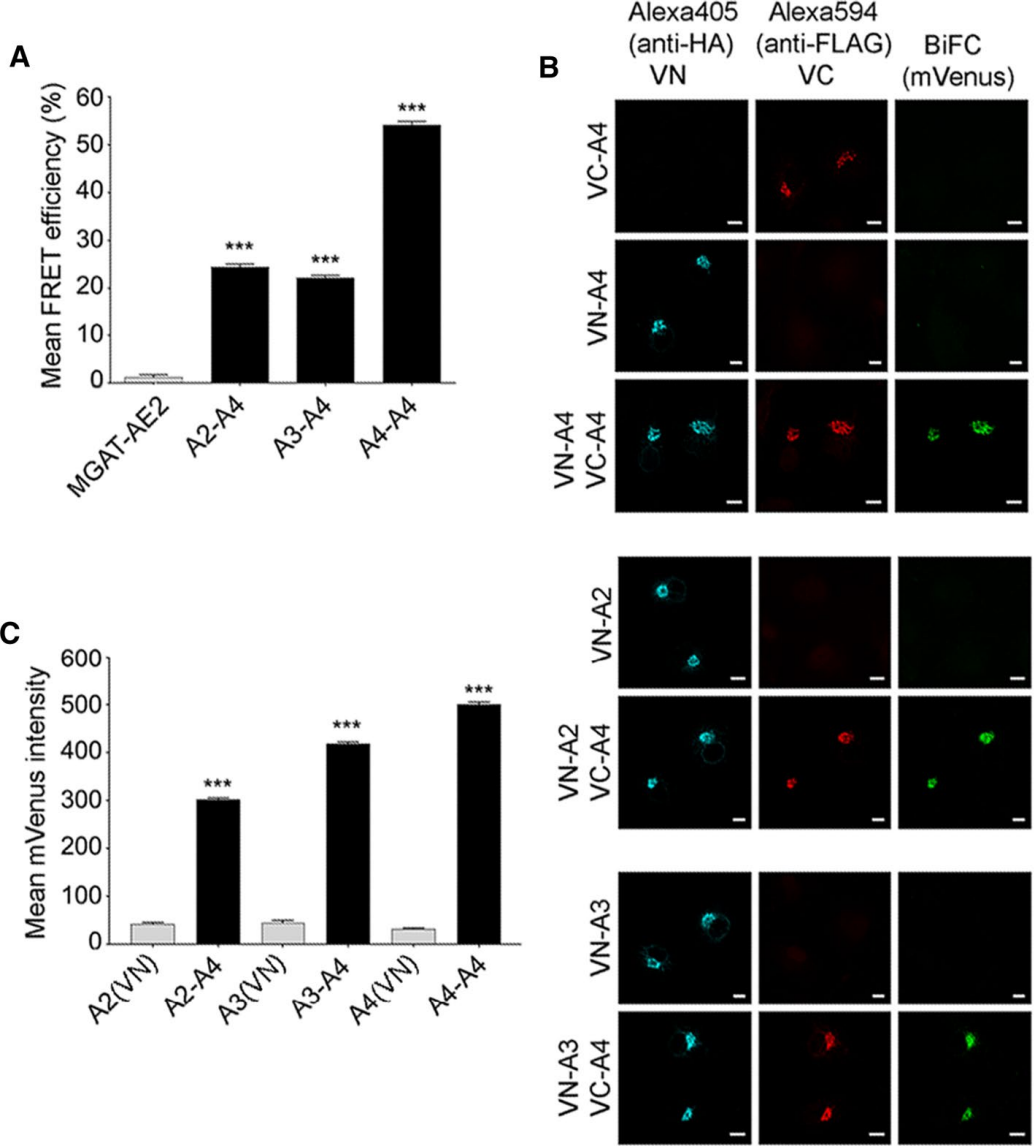
Interactions between the nucleotide sugar transporters SLC35A2, SLC35A3 and SLC35A4 (abbreviated A2, A3 and A4, respectively). **a** FRET analyses of A4-mVenus interactions with mCherry-A2, mCherry-A3 and mCherry-A4. The experiments were done mainly as described for Fig. 1, except that we used MGAT2-mVenus and mCherry-AE2 pair as a negative control. A statistical significance was assigned using one-way ANOVA with Tukey’s Post Hoc test (± SD, *n* = 3, ***p < 0.001). **b** BiFC analyses of the same interactions. After cell transfections with the depicted BiFC enzyme constructs, BiFC signals were quantified for both single (negative control) and double transfectants using Operetta High-Content Imaging System. Mean mVenus fluorescence signal was quantified in the Golgi region by averaging the signal from at least 10,000 VN and VC-positive cells. The bars denote data values after subtracting the mean mVenus fluorescence intensity in negative control areas (single transfectants) from those obtained with double transfectants. Statistical significance was calculated using one-way ANOVA with Tukey’s Post Hoc test, (***p < 0.001). **c** Immunofluorescence detection of the BiFC signal with a confocal microscopy. After the transfections, VC-positive cells were visualized using rabbit anti-FLAG primary antibody and anti-rabbit Alexa Fluor 594 secondary antibody (red) with an appropriate filter set. VN-positive cells were counterstained with mouse anti-HA primary antibody and anti-mouse Alexa Fluor 405 secondary antibody and specific filter set for Alexa 405 (cyan). BiFC signal was detected using the YFP filter set (green). Bars = 10 µm

To examine whether NSTs similarly form heteromeric assemblies in the Golgi membranes, we focused on interactions between the UDP-Gal transporter (SLC35A2, termed A2), the UDP-GlcNAc transporter (SLC35A3, termed A3) and SLC35A4 (termed A4), a putative NST due to its sequence similarity and ability to contribute to the sub-cellular distribution of an A2/A3 complex [36]. Co-transfection of COS-7 cells (Fig. 3a) with A4-mVenus together with either mCherry-tagged A2 (Golgi splice variant), A3, or A4 revealed in all cases an FRET signal that was clearly higher than that of the negative control MGAT2-AE2 (Fig. 3a). AE2, similar to NSTs, is a multi-spanning anion exchanger in the Golgi compartment that is not involved in nucleotide sugar transport across the Golgi membranes [13]. Therefore, the interactions between the NSTs are highly specific and not caused by overexpression of the constructs. Similarly to other NSTs, A4 also formed homo-oligomeric assemblies in the Golgi membranes of transfected cells [1, 14, 19, 27, 29, 31, 32].

To confirm the interactions, we utilized also the BiFC (bimolecular fluorescence complementation) approach. This method relies on the formation of a fluorescent mVenus protein from its non-fluorescent N- and C-terminal fragments (VN and VC) if brought close enough by interacting partners [11]. Therefore, we co-transfected COS-7 cells with the relevant plasmids and measured the mVenus-BiFC signal in the Golgi region of single- (used as a negative control) and double-transfected cells. Selection of the Golgi regions of interest (ROI) was done after immunofluorescence staining of the fragments with epitope-tag-specific antibodies (see Fig. 3b). We found that all the tested combinations (A4/A4; A2/A4, A3/A4) gave a strong BiFC signal in the Golgi region of double-transfected (both VN and VC) cells, but not in single transfected cells (either VN or VC alone). Quantitation of the signal intensities with the Operetta High-Content Imaging System showed that the BiFC signal intensity of the A4 homodimers and heterodimers with A2 or A3 were significantly higher compared to cells expressing only the N-terminal fragment of the mVenus protein (Fig. 3c). Thus, both our FRET (Fig. 3a) and BiFC (Fig. 3b, c) data show that the NSTs, similarly to MGATs, form heteromeric assemblies in Golgi membranes in vivo.

### Direct detection of ternary complexes using a BiFC-based FRET approach

**Fig. 4:**
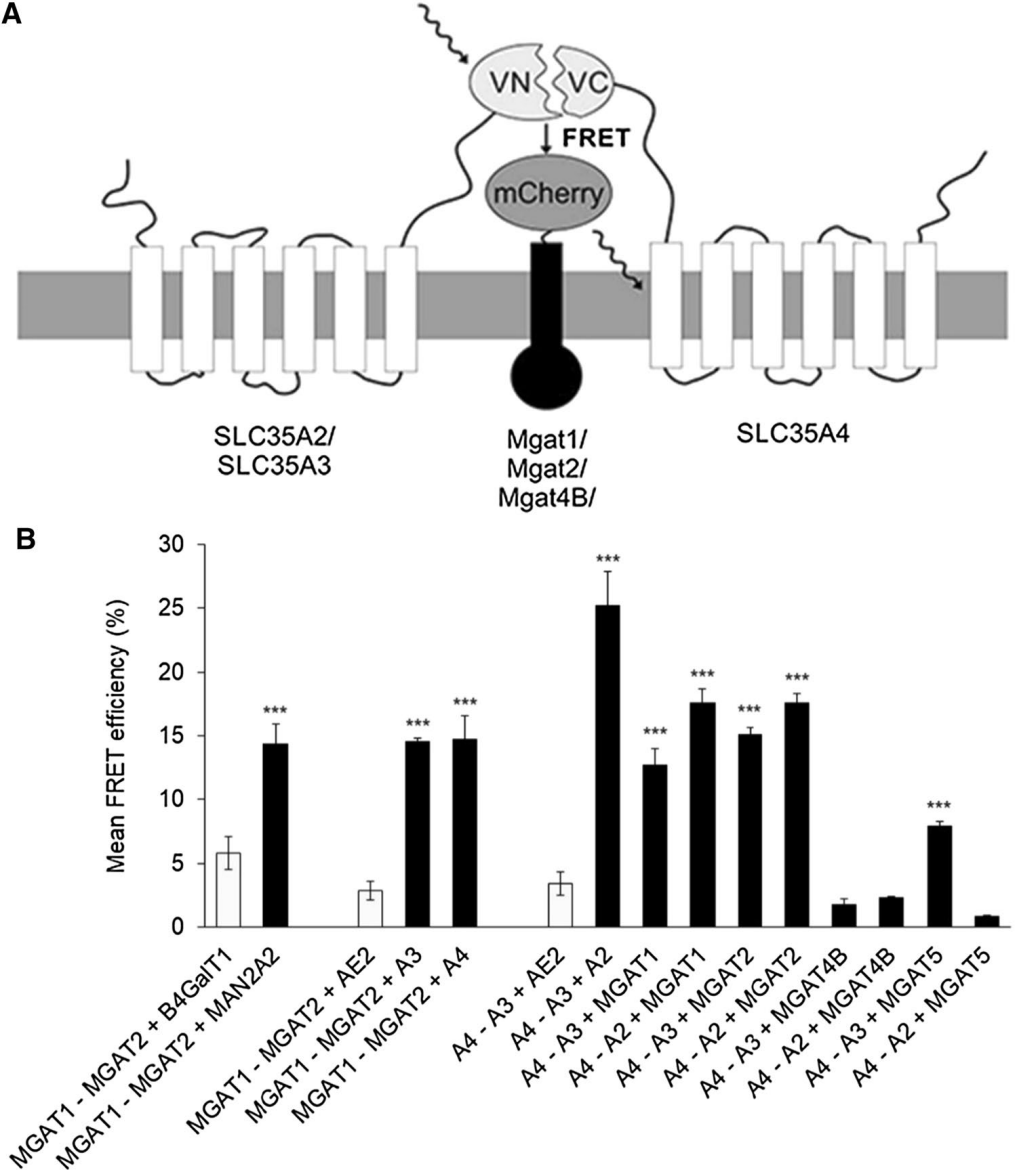
Identification of ternary complexes with BiFC–FRET approach**. a** Schematic representation of BiFC-based FRET approach adopted here detecting the ternary complexes between the medial Golgi enzymes, the NSTs and the mutual interactions between the MGATs and NSTs. **b** BiFC–FRET analyses of the depicted enzyme and transporter constructs. Cells were triple transfected with the constructs, fixed 24 h later before quantification of the FRET signal using the Operetta High Content Imaging System. As negative controls (light grey columns) we utilized different combinations, each of which contained one non-interacting construct (B4GALT1, AE2). Statistical significance of the changes was calculated using one-way ANOVA with Tukey’s Post Hoc test (± SD, *n* = 3, ***p < 0.001)

A BiFC-based FRET approach has previously been used to detect ternary complexes between cytoplasmic and nuclear proteins [12, 17, 21, 35]. Here, we utilized this approach (Fig. 4a) to directly verify potential ternary interactions (Figs. 3 and 4) between the MGATs, between the NSTs themselves, or between MGATs and NSTs. To accomplish this, we triple-transfected COS-7 cells with two BiFC MGAT or transporter constructs together with one mCherry-tagged or mCerulean-tagged enzyme or transporter construct (Fig. 4a). As a negative control, we utilized mCherry-tagged AE2 anion exchanger construct. All the constructs were designed to have similar membrane topology. Thus, for the enzyme–transporter interactions the tags were placed on the cytosolic surface of Golgi membrane, while in the enzyme–enzyme experiments the tags were facing the Golgi lumen (Fig S1). Quantification of the BiFC–FRET signals with the high-content imaging system revealed several distinct ternary complexes between the test proteins. One of these consists of MGAT1, MGAT2 and MAN2A2 (Fig. 4B), as expected based on their known mutual interactions (see Figs. 1 and 2). We also identified ternary complexes between the three transporters A4, A3 and A2. Replacement of A2 in the assay with the AE2 anion exchanger construct reduced the signal markedly, confirming that the interactions between the three transporters are specific. Moreover, we detected simultaneous ternary interactions between two MGATs and one transporter (Fig. 4b), including those between MGAT1, MGAT2 and A3, or between MGAT1, MGAT2 and A4. We also detected simultaneous ternary interactions between two transporters and one MGAT, including those between A3, A4 and MGAT1 (or MGAT2), or between A2, A4 and MGAT1 (or MGAT2). However, MGAT5 did not interact with A4 and A2 transporters (Fig. 4b) but did so with A4 and A3 transporters, suggesting that A3 provides the substrate for MGAT5. Intriguingly, we did not detect ternary complexes between MGAT4B and the NST pairs investigated, suggesting that MGAT4B interacts with an unidentified transporter for getting its donor substrate for catalysis. Collectively, these data provide the first direct evidence for the existence of ternary complexes between the MGATs and the NSTs, as well as complexes between them. The data thus reveals the existence of distinct multi-enzyme–transporter assemblies in the Golgi membranes in vivo.

## Discussion

Previous work on medial-Golgi MGATs had established heteromeric interactions separately only between MGAT1 and MGAT2 or MGAT1 and MGAT2 with MAN2A2 in the ER [24, 25]. Maszczak-Seneczko et al. [18–20] had also shown interactions between the SLC35A3 and SLC35A2 transporters [19] or between the MGAT1, MGAT2, MGAT4B or MGAT5A and the transporters [18, 20]. Our findings show for the first time multiple and simultaneous interactions between the MGATs themselves and between the MGATs and NSTs. For example, our in vivo interaction and inhibition studies in the Golgi apparatus revealed several distinct MGAT complexes (Fig. 2e), including the ones between MGAT1, MGAT2 and MAN2A2, between MGAT1, MGAT4B and MAN2A2, or between MGAT2, MGAT3 and MAN2A2. Based on these data (Figs. 1 and 2), we suggest that MAN2A2 serves as a central hub for the MGAT interactions, as it can bind, in a competitive manner, to three different MGAT sub-complexes (MGAT1–MGAT2, MGAT2–MGAT3 and MGAT4B–MGAT1). Therefore, MAN2A2 seems either to have only one binding interface for each of these three MGAT sub-complexes. Alternatively, it may have separate but interdependent binding interfaces for each of them, in which the binding of one sub-complex modulates the other interface so that another sub-complex cannot simultaneously bind to MAN2A2. In either case, however, the MGATs were able to form ternary complexes with MAN2A2. This was also confirmed using the BiFC–FRET approach (Fig. 4b). The specificity of the interactions was confirmed in each case using negative controls, indicating that the interactions are not driven by overexpression of the constructs. In further support, some of the interactions including the one between MGAT1 and MGAT2 have been detected earlier by other means [24, 25].

From the functional point of view, our current data, however, do not allow to discern how these sub-complex assemblies function in *N*-glycan processing. If the assemblies are static, the acceptor *N*-glycan is then expected to shuttle between the different sub-complexes to undergo stepwise processing, in which case each sub-complex should recognize the acceptor separately. This may be mediated by MAN2A2 as it is present in all sub-complexes detected in our MGAT interaction screens. Therefore, it may act as a central hub being able to recruit other enzymes and the acceptor substrate that needs further processing. In this case, it is curious that we did not identify any interaction partners for MGAT5, suggesting that it must recognize its acceptor by itself. An alternative possibility also is that the various sub-complexes (i.e. MGAT1, MGAT4B and MAN2A2 or MGAT2, MGAT3 and MAN2A2) may process distinct *N*-glycan precursors that differ in the number of the antennae added to the high mannose *N*-glycan. In this scenario, the function of the MGAT1/MGAT2/MAN2A2 complex would thus be to synthesize only biantennary *N*-glycans, while the others would be involved in making tri- or tetra-antennary *N*-glycans. A third possibility exists and may involve dynamic exchange of sub-complex constituents. In this case, the acceptor may stay bound to the primary complex (MAN2A2–MGAT1–MGAT2), whose composition then gradually changes by replacement of complex constituents with other enzymes. For example, MGAT1 in the primary complex could be replaced by MGAT3 (or MGAT3–MGAT2 sub-complex) that then adds the bisecting *N*-acetylglucosamine to an already partly processed *N*-glycan. Likewise, MGAT4B (or MGAT4B–MGAT1 complex) that adds GlcNAc at C-4 of the core α1-3Man to yield another tri-antennary *N*-glycan could replace MGAT2 (or MGAT2–MGAT1 complex, respectively) in the primary ternary complex. How such replacements between the complex constituents are coordinated during the processing steps remain unclear but may depend on each GlcNAc residue added to an *N*-glycan.

**Fig. 5:**
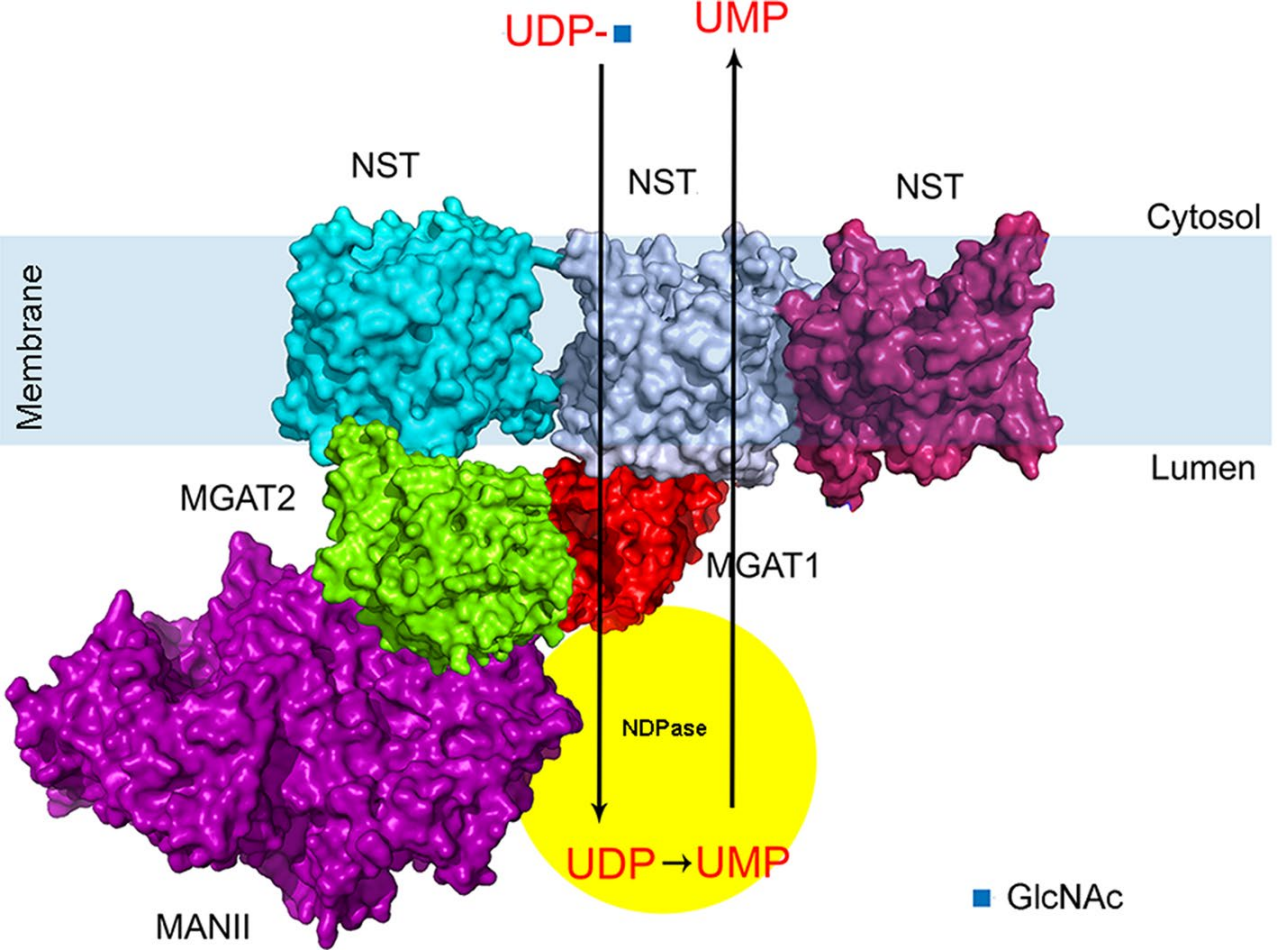
A schematic model of the multi-enzyme/multi-transporter assemblies in the Golgi membranes of live cells. The interactions were deduced from the data presented in Figs. 1, 2, 3 and 4. The model represents the primary complex consisting of MGAT1, MGAT2 and MANII (MAN2A2). The complex is shown to import UDP-*N*-acetylglucosamine (UDP-GlcNAc) into the Golgi lumen, where after MGAT1 is adding the sugar to acceptor (not shown) and freeing UDP. UDP is then transformed to UMP by the nucleotide diphosphatase (shown as part of the same assembly). UMP then drives the import of a new UDP-GlcNAc residue for further catalysis. The structures shown were obtained using existing atomic coordinates present in the PDB database. The following ID numbers were used: 2am3 (MGAT1), 5vcm (MGAT2), 3czs (MAN2A2) and 5ogk (NSTs)

Our findings that MGATs and NSTs also show interactions are important as they expand our understanding on Golgi membrane organization in the living cell. Firstly, the data shown are compatible with previous pull-down studies, which showed the presence of huge mega-dalton size medial-Golgi enzyme complexes in cells [28]. In addition, the simultaneous ternary interactions among the MGATs themselves or with the NSTs shown here indicate for the first time that such multi-enzyme–transporter complexes can exist in live cells. This means also that each member of such an assembly must have more than one interaction surface to bind two or more partners simultaneously. We demonstrated this by showing that, e.g. MGAT3 binding to MGAT2 did not inhibit MGAT2–MAN2A2 interaction. Secondly, our data suggest the existence of enzyme–transporter assemblies that could couple sugar transport to its simultaneous attachment to a protein, lipid or an immature glycan. This possibility is intriguing and adds to the current view stating that the main role of the transporters in glycosylation is to concentrate nucleotide sugars in the Golgi lumen and facilitate their use as donors for the catalysis. However, since each monophosphate is the end product of sugar addition and needs to be exported back to the cytosol in exchange for another nucleotide sugar import, it seems that such concentration in the Golgi lumen of nucleotide sugars by the transporters is unlikely. In contrast, by functionally coupling enzymes with their relevant transporters, such concentration may not be needed, as the sugar can be transferred directly from the transporter to the enzyme in the same complex (Fig. 5). In this scenario, transport of the monophosphates back to the cytosol would require simultaneous import of the UDP-sugar before and export of monophosphate by the same transporter. Thus, such enzyme–transporter assemblies would suffice for efficient glycan synthesis. However, further studies are needed to directly prove whether this hypothesis is correct and awaits the demonstration of whether the absence of such assemblies causes glycosylation defects. A prerequisite in these studies is identification of interaction surfaces to allow mutagenesis studies aimed at inhibiting complex formation in vivo.

We also noticed that MGAT1/2 interact not only with the respective transporter, SLC35A3, but also with SLC35A2 and SLC35A4. It is currently unclear why MGATs interact with the latter two, given that the former is a galactose transporter and the latter is an unknown transporter with no substrate sugar identified yet. Yet, one possibility that may explain these interactions is that the sugar specificities of the transporters may not be absolute [23] and might be regulated by the enzymes attached to them. Alternatively, it is important to realize (particularly in the case of the A2 transporter) that the interactions are mediated by their transmembrane and/or cytosolic domains (in contrast to enzyme interactions that are mediated mainly by their catalytic domains). This suggests that this interaction is not functionally relevant for the catalytic activity of MGAT1 or MGAT2. Rather, we think that this interaction may relate to acceptor “channeling” to the next enzyme (B4GALT1), a phenomenon that may require later enzyme (B4GALT1) recycling along with its A2 transporter to an earlier compartment, as the Golgi maturation model assumes. Thereby, A2/B4GALT1 complex can become close to MGAT’s TM and cytosolic domains, helping B4GALT1 to retrieve the acceptor from the MGAT1’s catalytic domain for further processing. After giving up the acceptor, MGAT1/2 would be able to recycle into the cis-cisternae and turn it to a new medial Golgi compartment. In our experimental system, such delivery of the acceptor likely does not happen due to the lack of acceptor molecules relative to overexpressed enzyme present, whereby the A2/MGAT1/MGAT2 complexes keep accumulating. Further work is needed to test whether this scenario is correct or not.

To summarize, the data described in this report provide direct evidence for the existence of several multi-enzyme/multi-transporter complexes in Golgi membranes. Such assemblies likely result from the tendency of the GTases and the NSTs to self-assemble with each other, explaining the co-existence of different NSTs with the same nucleotide sugar specificity, as each one of them could deliver the nucleotide sugar only to a distinct enzyme or set of enzymes. Such assemblies are also functionally important as they seem to facilitate more efficient synthesis of complex type *N*-glycans in the medial-Golgi [15]. In addition, the ability of the GTases and the NSTs to self-assemble into higher order oligomers may also serve not only glycosylation but also Golgi residency, Golgi membrane organization and Golgi stack morphology. It is anticipated that such assemblies also include other Golgi resident proteins including a phosphodiesterase and perhaps proteins associated with Golgi membrane trafficking. These data thus form the basis for future studies aimed at defining all the players in these Golgi membrane enzyme–transporter assemblies and understanding how their formation and functions are regulated at the level of Golgi membranes in living cells.

## Electronic supplementary material

Below is the link to the electronic supplementary material.

Supplementary material 1 (DOCX 851 kb)
