# Supplementary material for: N-acetylglucosaminyltransferases and nucleotide sugar transporters form multi-enzyme–multi-transporter assemblies in golgi membranes in vivo
Source: Cell Mol Life Sci. 2019 Feb 8;76(9):1821–32. doi: 10.1007/s00018-019-03032-5 (PMC6453868; doi:10.1007/s00018-019-03032-5)

**Supplementary information:**

**Supplemental figure legends**

**Fig. S1**. Plasmids employed in BiFC and FRET interaction screens. Enzymes were tagged either N-teminally or C-terminally, while the transporters were tagged only N-terminally. The BiFC pair is VN-VC in all cases and the partner FRET pair is mCerulean for enzymes and mCherry for transporters.

**Fig. S2**. Co-localization of the MGAT constructs with the Golgi marker by fluorescence microscopy. Cells were transfected with the depicted mVenus constructs and 24 h later, fixed and counterstained with the cis-Golgi marker anti-GM130 antibodies. Optical sections were taken by confocal microscope using appropriate filter set for mVenus and the goat anti-mouse secondary antibody used for visualization of the bound mouse anti-GM130 monoclonal antibody. Bars 10 µm.

Fig. S1


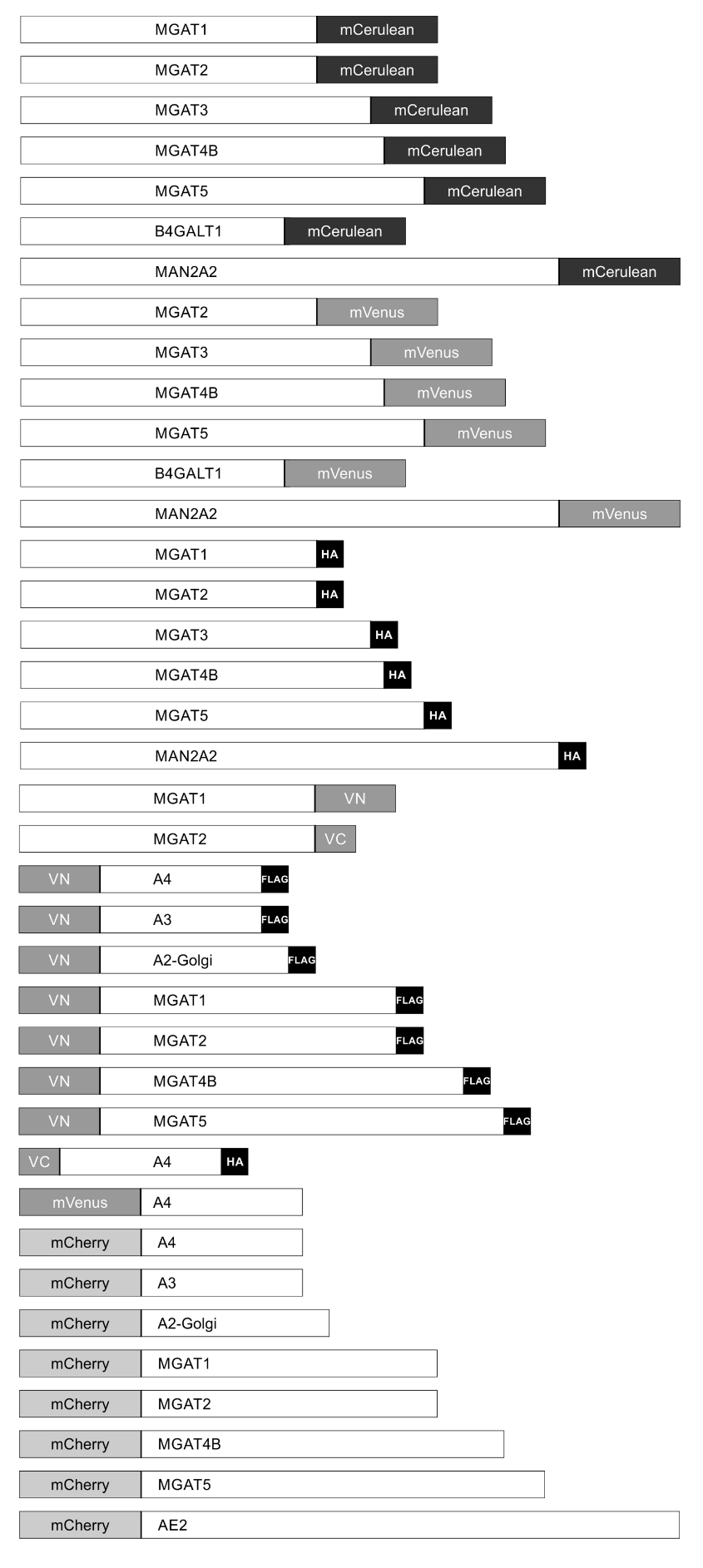


.Fig. S2


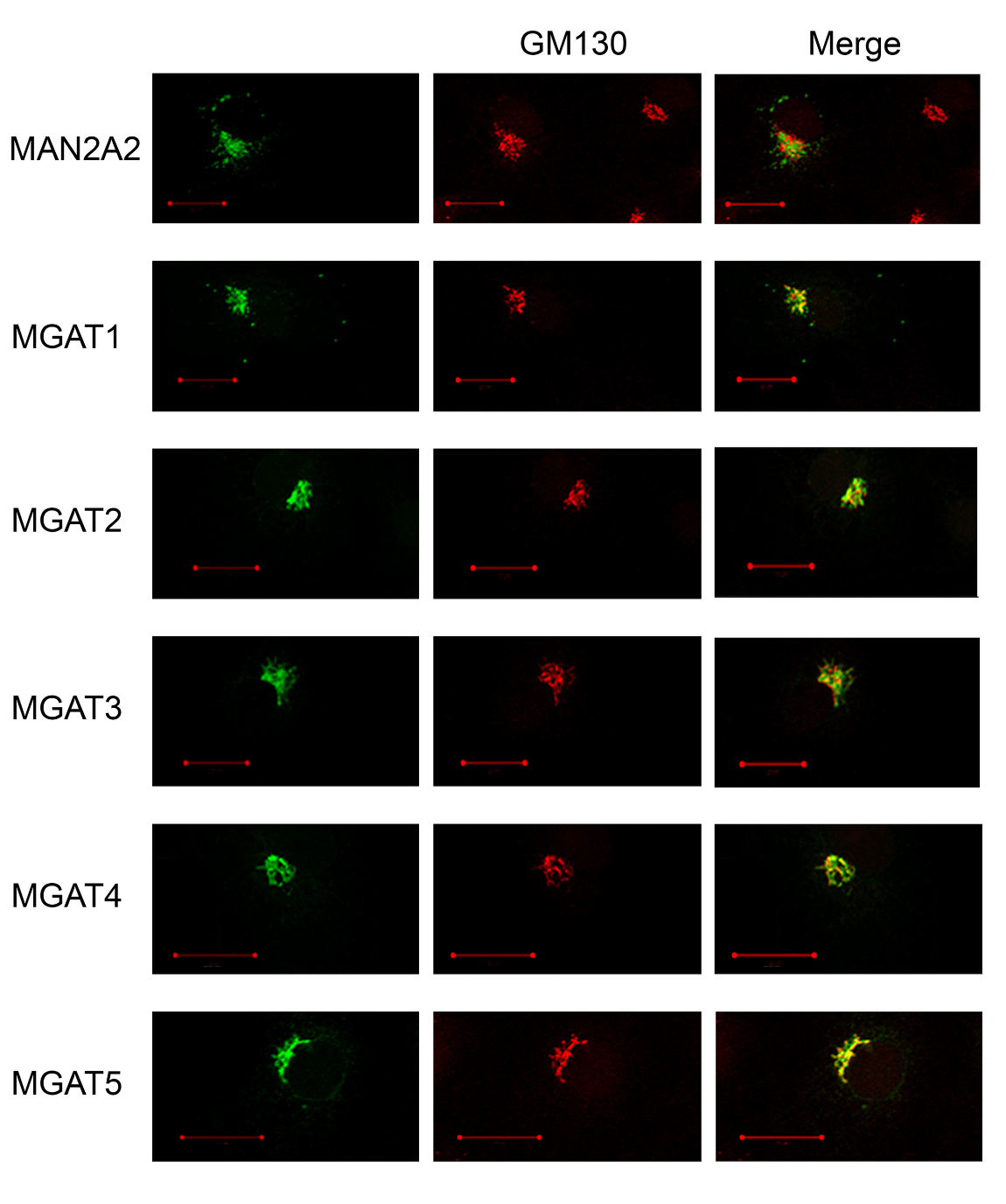

Supplement: Supplementary file 1 — Supplementary material 1 (DOCX 851 kb) [file 18_2019_3032_MOESM1_ESM.docx]
